# Diagnostic role of DOG-1, GFAP and B-catenin in Basal cell Adenoma and Cellular Pleomorphic Adenoma of the Salivary Gland

**DOI:** 10.1007/s12105-022-01498-7

**Published:** 2022-10-28

**Authors:** Álvaro López-Janeiro, Luis Blasco-Santana, Manuel Pérez-Pérez, Elena Ruiz-Bravo

**Affiliations:** 1 Department of Pathology, Clínica Universidad de Navarra, Av. de Pío XII 36, Pamplona, Navarra Spain; 2 Department of Pathology, Hospital Infantil Universitario Niño Jesús, Madrid, Spain; 3 Department of Pathology, Hospital Universitario Virgen de Valme, Seville, Andalusia Spain; 4 Department of Pathology, University Hospital La Paz, Madrid, Spain

**Keywords:** Basal-cell adenoma, Pleomorphic Adenoma, Immunohistochemistry

## Abstract

**Background:**

Pleomorphic Adenoma (PA) and Basal cell adenoma (BCA) are benign salivary gland tumors that may pose a diagnostic challenge if typical features are not present. Due to the increased relapse and malignant transformation rate of the former, a correct diagnosis carries relevant prognostic information. Even though immunohistochemistry (IHC) plays a limited role in the diagnosis of these tumors, the use of IHC panels could increase diagnostic accuracy. In the present work, we aimed to demonstrate that the use of an IHC panel consisting of Glial Fibrillary Acid Protein (GFAP), B-Catenin and Discovered On GIST 1 (DOG-1) can aid in the differential diagnosis between PA and BCA.

**Methods:**

We analyzed 18 cases of benign salivary gland tumors (Pleomorphic adenomas and Basal cell adenomas) with overlapping histologic features. First, a head and neck pathologist diagnosed the cases relying on morphology alone. Afterwards, cases were re-evaluated considering the IHC panel results. Inter-observer IHC scoring concordance was evaluated with pre-defined marker cut-off points using Cohen’s Kappa scores.

**Results:**

Based on morphology alone, 9 cases were classified as PA while the remaining tumors were considered to be BCA. Five out of nine BCA cases showed GFAP staining and absent nuclear B-catenin and DOG-1 positivity. Conversely, 2 PA cases showed absent GFAP and positive nuclear B-catenin with concurrent DOG-1 expression. Therefore, after IHC evaluation, up to 40% of morphologic diagnoses were reconsidered. Overall, the inter-observer concordance for IHC evaluation was good (resulting Kappa Scores between 0.78 and 1).

**Conclusion:**

Our work supports the use of a concise IHC panel to improve the diagnostic accuracy of benign salivary gland tumors with overlapping histologic features.

## Introduction

The diagnosis of salivary gland tumors with typical features is usually straightforward, even for the unexperienced pathologist. However, it is well known that salivary gland tumors with overlapping morphology represent a diagnostic challenge [1–3].

Pleomorphic Adenoma (PA) is the most frequent salivary gland epithelial tumors in both adults and children. Most occur in the parotid gland, typically in the superficial lobe. When intra-oral they tend to occur in the hard palate [4, 5]. Women are more frequently affected than men are. The mean age at diagnosis is around 45 years [6]. Aside from relapsed tumors, most PA present as single masses. Macroscopically PA is well defined, usually demonstrating a thin non-constant capsule. Grayish cartilage-like or myxoid areas can be identified on gross analysis. PA may also show cystic degeneration, especially if fine needle aspiration of the mass has been performed before resection. Under the microscope, a double population of epithelial and myoepithelial cells compose this tumor. Characteristically, fibrous, chondro or myxoid stroma can be identified. However, PA show high morphology variation, and a pleiotropy of morphologic presentations can be found [4–7].

Although surgery is the mainstay of treatment, variable surgical approaches are currently recommended [8, 9]. Relapse rates reach 2.9% and are usually attributed to incomplete resection, intra-operative tumor spillage or excessive tumor manipulation. Although rare, tumor malignant transformation is possible. Transformation risk is higher in long evolving tumors and is associated with patient’s age [10]. Even if PA is considered a benign tumor, metastases have been reported [11].

Basal cell adenoma (BCA) is a rare entity that represents 1-3.7% of primary salivary gland tumors [6]. It also occurs more frequently in the parotid gland, with the lip being the most frequent intra-oral location [1, 6]. It also demonstrates female predominance. Compared to PA, it usually occurs in slightly older patients (mean presenting age of 50 years) [6]. Except for the membranous subtype, most BCA cases are macroscopically well circumscribed. Microscopically, the tumor is composed of a biphasic population of basaloid appearing epithelial and myoepithelial cells. The characteristic stroma of PA is absent. Several growth patterns are recognized, including tubulotrabecular, cribriform, membranous and solid [6]. Peripheral palisading with thick basal membrane is commonly seen. Apart from the membranous subtype, relapse after surgery is exceedingly rare.

PA and BCA show histological overlap, most commonly when occurring in the minor salivary gland. In this location, the characteristic PA stroma is focal or absent, and tumors show a more cellular appearance [12]. This can pose a diagnostic challenge. Even though, morphology remains the diagnostic gold standard, an IHC panel could further improve accuracy when facing this diagnostic dilemma. In addition, this specific diagnostic challenge has not been adequately addressed previously in literature.

In this study we have evaluated the IHC expression profile of Glial Fibrillary Acid Protein (GFAP), B-Catenin and Discovered On GIST 1 (DOG-1) on cellular PA and BCA. Although previously published work has demonstrated the expression of GFAP in PA and the expression of DOG1 and nuclear B-Catenin in BCA[13, 14], an immunohistochemical panel has not been tested to differentiate between cellular PA and BCA. In addition, a panel including several marker will potentially achieve higher sensitivity and specificity.

## Methods

The electronic records of University Hospital La Paz were screened to find cases categorized as PA, BCA or basaloid benign primary salivary gland tumor. Only surgical resection specimens were included in the present study. Tissue slides were reviewed by a pathologist, discarding cases with evident chondromyxoid stroma, and selecting highly cellular tumors with overlapping morphologic characteristics between PA and BCA. Solid and trabecular BCA were included in the present study. BCA with membranous pattern cases were not considered in the present work. Follow-up information was obtained through the electronic clinical records. Afterwards, an expert head and neck pathologist was asked to morphologically review a single representative hematoxylin and eosin-stained slide from each of these cases. The expert pathologist classified the tumors on morphologic ground alone into PA or BCA.

Afterwards, three-micron thick tissue sections from formalin fixed paraffin embedded tissue blocks were used to perform immunohistochemistry. Immunohistochemistry was performed on a Dako OMNIS auto-stainer (Agilent) following manufacturer instructions. Primary antibodies against DOG-1 (SP31, Genova, 1:100), GFAP (Polyclonal, Dako-OMNIS, prediluted), and B-catenin (B-catenin-,1 Dako-OMNIS, prediluted) were used. EnVision™ system was used as secondary labelling system. After immunostaining, samples were counterstained with Harris Hematoxylin for 7 min followed by PBS rinsing. Slides were mounted using non-aquous medium. Healthy salivary gland tissue present in samples served as positive internal control for DOG-1 (apical staining in acini) and B-Catenin (membranous staining in acini). Brain cortex was used as external positive control for GFAP immunohistochemistry.

To calculate inter-observer concordance, immunohistochemistry was evaluated by two independent pathologists according to the following criteria:

- DOG1 was evaluated according to the presence or absence of cytoplasmic or membranous (apical-luminal, basolateral or complete) staining, regardless of the cell-type (epithelial, myoepithelial or stromal) stained. Taking as reference the staining of healthy acini (2+), IHC intensity in tumor cells was scored as faint (1+), moderate (2+) or intense (3+). Percentage of positive immune cells was semiquantitatively evaluated across 5 high power fields (40x) (1+: <5% of tumor cells positive, 2+: 5–50% of tumor cells positive, 3+: >50% of cells positive) according to previously published work [15].
- B-Catenin was evaluated according to previous studies [16–18]. Nuclear expression in at least 3% of tumor cells (either epithelial or myoepithelial) was considered positive. Membranous or cytoplasmic staining was disregarded.
- The presence of tumor cells stained with GFAP was scored, independent of staining pattern and intensity as reported in past research studies [13]. In addition, staining was scored regardless of the cell type (epithelial, myoepithelial or stromal). GFAP expression was dichotomized using an arbitrary 10% cut-off point to stratify tumors into those demonstrating absent/very focal positivity from tumors demonstrating diffuse GFAP expression.

Kappa scores were calculated to evaluate inter-observer agreement. DOG1 assessment was dichotomized into present or absent. B-catenin and GFAP were dichotomized as described above. Kappa scores and 95% confidence intervals were calculated using cohens.kappa function from *psych* R package (version 2.1.6).

After IHC evaluation, both pathologists reviewed the initial diagnosis and made a definitive integrated diagnosis.

## Results

18 cases were included in our analysis (Table 1). Based on morphologic findings, 9 cases were diagnosed as PA and 9 were diagnosed as BCA by the expert pathologist. Twelve of the samples belonged to female patients (67%) and the mean age at diagnosis was 59 years (range 34–78 years). Most tumors (15/18, 83%) were located in the parotid gland.

**Fig. 1:**
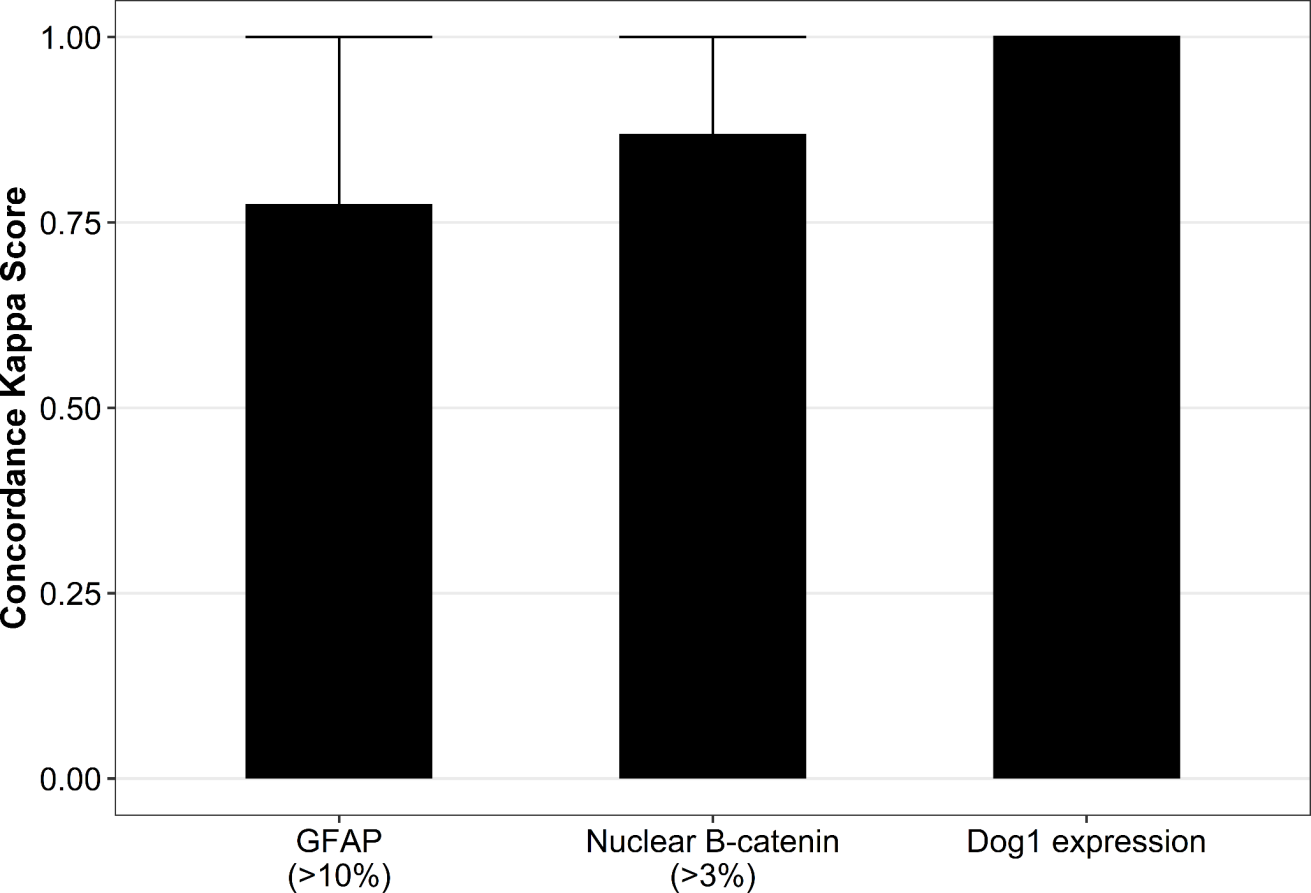
Bar plot showing concordance Kappa scores and 95% CI for each immunohistochemical marker. Since DOG1 showed perfect concordance confidence intervals could not be calculated

For all immunohistochemical markers high inter-observer concordance was observed (Fig. 1). Notably, DOG1 evaluation showed perfect concordance (kappa score = 1). GFAP and B-catenin showed almost perfect concordance (Kappa scores 0.78 and 0.87, respectively).

Focal or diffuse GFAP staining was observed in 11 out of the 18 cases analyzed. GFAP staining was identified in periductal and mesenchymal like fusiform cells that are normally adjacent to epithelial nests. Seven cases showed complete absence or only minimal GFAP staining. Interestingly, 5 cases receiving a morphologic diagnosis of BCA showed GFAP positivity with a similar pattern. In addition, none of these BCA samples demonstrated nuclear B-catenin staining. Six cases harbored at least 3% of nuclear B-catenin positive tumor cells. In these positive cases, nuclear B-catenin positivity was restricted to myoepithelial cells. Four of these cases received a morphologic diagnosis of BCA and the remaining two were diagnosed as PA. All other cases showed membranous B-catenin staining. DOG-1 cytoplasmic and membranous expression was found in 6 cases. All positive cases harbored at least 5–50% of positive tumor cells. In addition, all positive cases showed an average moderate to intense staining intensity (2+/3+). Most stained cells were located on the tumor periphery, probably reflecting myoepithelial cell staining. Importantly, all cases demonstrating DOG-1 positivity co-expressed nuclear B-catenin. Only a single case demonstrated complete absence of stain for the three immunohistochemical markers.

**Fig. 2:**
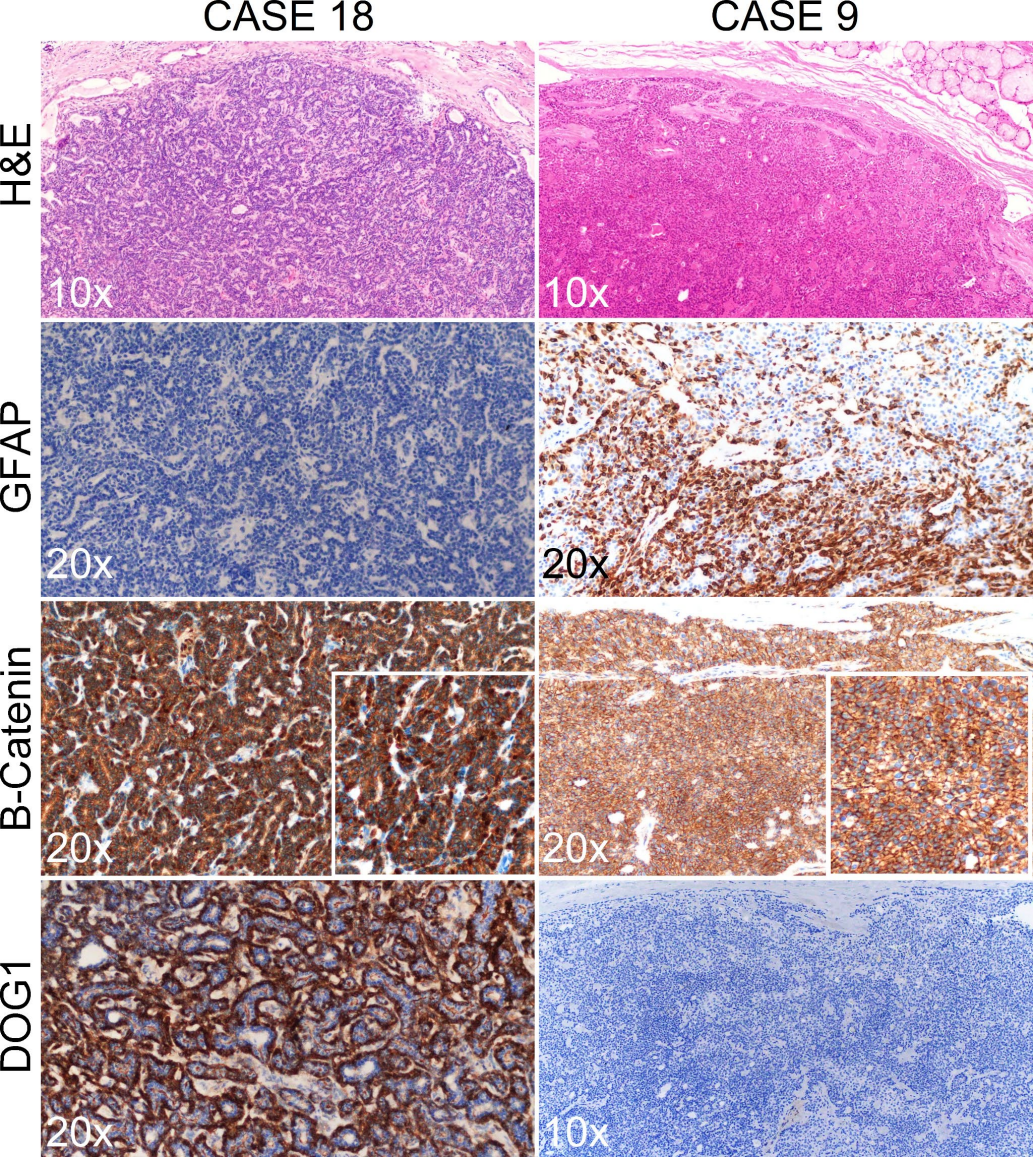
Representative images from cases 18 and 9. Case 18 was initially diagnosed as Pleomorphic Adenoma and reclassified as Basal cell Adenoma. Case 9 was originally diagnosed as basal cell adenoma and reclassified as pleomorphic adenoma

**Fig. 3:**
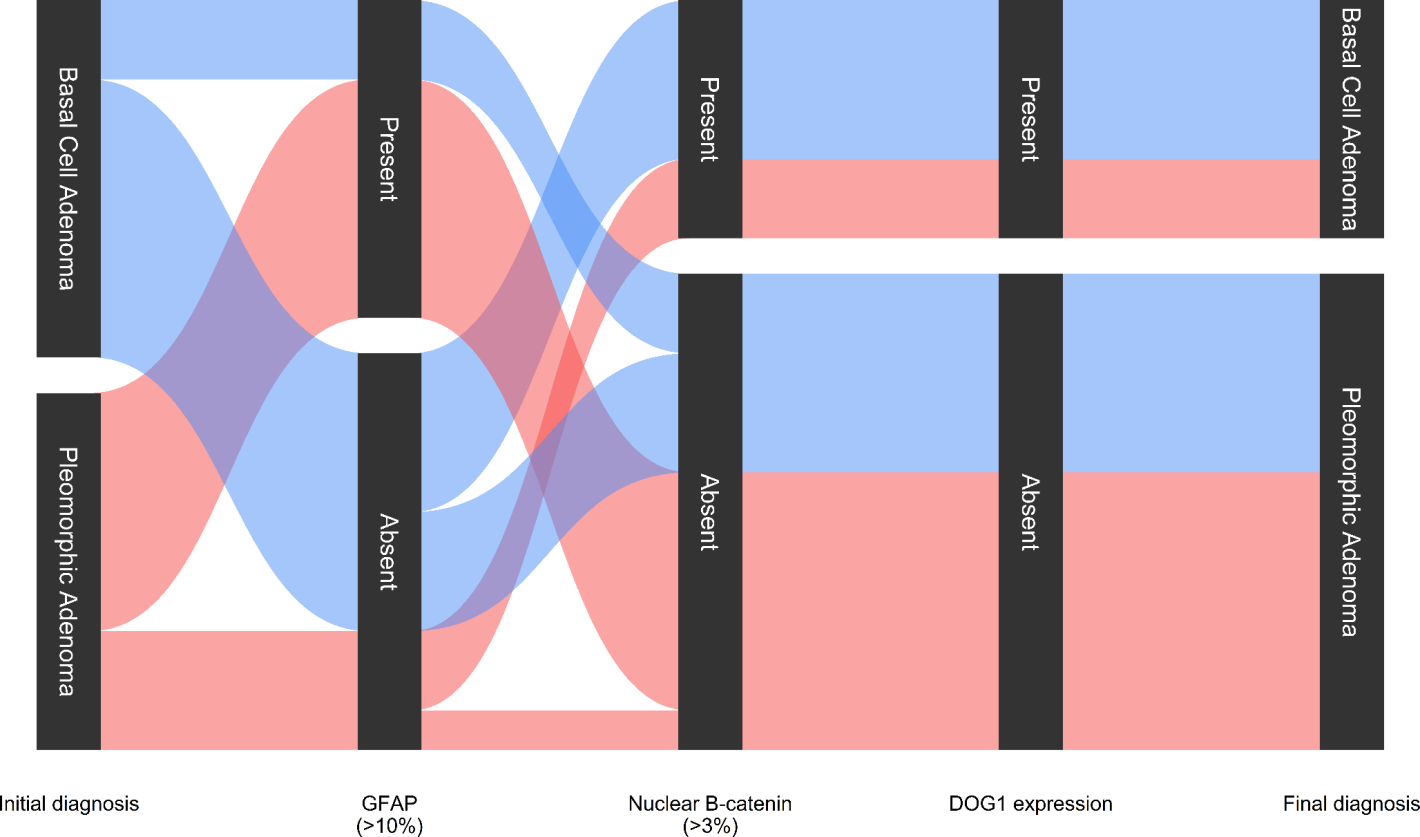
Parallel set plot denoting diagnostic flux through immunohistochemical evaluation. Blue connector lines represent cases initially diagnosed as basal cell adenomas and red connector lines represent cases initially diagnosed as pleomorphic adenomas

Taking into account the high sensitivity and specificity of nuclear B-catenin (applying a cut-off point of 3% stained tumor nuclei) in BCA and its low expression in PA according to published literature[14, 17, 18], we reevaluated the morphologic diagnoses in the light of the IHC panel results (Figs. 2 and 3). As depicted in Fig. 3, all PA cases that expressed nuclear B-catenin concurrently expressed DOG1 (at least 5–50% of positive tumor cells) and were negative for GFAP. Conversely, 5 BCA cases showed positive GFAP staining and absent nuclear B-catenin and DOG1. The flowchart in Fig. 3 denotes the high correlation between immunohistochemical markers, as all tumors showing nuclear B-catenin positivity also stained with DOG1. On the other hand, all GFAP positive tumors and a single GFAP negative tumor consistently scored negative for B-catenin and DOG1 IHC (Fig. 3). Therefore, we reclassified 7 cases after IHC evaluation (40% of cases). Five BCA were finally considered to be PA and 2 PA were reclassified as BCA. No relapses were recorded in the 12 patients that had available follow-up information. However, Case nº2, which was reclassified as PA after evaluating the IHC pattern, was a relapse from a tumor that was originally diagnosed as BCA.

## Discussion

Basaloid salivary gland tumors are a heterogeneous group of benign and malignant entities characterized by small cells with round to oval nucleus and scant cytoplasm. The benign basaloid tumors include PA, BCA, myoepithelioma and canalicular adenoma. When the key morphological characteristics of tumors are absent, the differential diagnosis may be problematic.

PA is the most frequently diagnosed salivary tumor. There is a risk of tumor relapse and up to 5–15% of cases may transform into a malignant tumor. On the contrary, BCA only rarely recurs (except for the membranous variant) and malignant transformation has not been reported [10]. Therefore, differentiating between both entities is mandatory. Up to 57.1% of PA demonstrate a basaloid pattern and 9.5% show a palisading architecture. Therefore, a subset of PA resembles BCA morphologically. In addition, the characteristic chondroid, myxoid or chondromyxoid stroma of PA may be scant or absent, mostly when PA is located in minor salivary glands [6].

Glial Fibrillary Acid Protein (GFAP) is an intermediate filament expressed in glial tissue. It has been previously reported to be expressed in periductal and mesenchymal cells of PA [12, 13]. The expression of GFAP in early myxoid and chondroid differentiation was first reported by Toshiro et al. In contrast, GFAP expression has not been reported in healthy salivary gland or other tumors, including BCA [19].

The use of DOG1 as a marker of acinar cells and intercalated ducts as well as a marker of acinar cell carcinoma has been previously explored [20, 21]. In the present study, we have demonstrated the expression of this marker in the myoepithelial cells of BCA, being particularly useful to differentiate cellular PA from BCA. Although other studies have reported the presence of variable DOG1 expression in PA [20–22], in the present study we have seen its expression to be tightly associated with BCA. Discrepancies could be explained by the diversity of clones used in our and previous studies. In addition, the pattern of myoepithelial cell DOG1 expression found in BCA in our work and other previously published studies [21] contrasts with the luminal staining pattern of other salivary gland tumors [20]. This finding is supported by previous studies demonstrating DOG1 expression in myoepithelial cells[21].

Nuclear B-catenin IHC expression has been reported in the myoepithelial cells of BCA and basal cell carcinoma [14, 17, 18]. A cut-off point of 3% of tumor cells with nuclear expression has been proposed by Masanobu Sato et al. According to their scoring criteria, a sensitivity of 94% and a specificity of 95% could be achieved for the diagnosis of BCA. Other studies have reported variable sensitivity (between 82% and 98%) of nuclear B-catenin for the diagnosis of BCA [14, 23] these findings should be interpreted taking into account that nuclear B-catenin expression is higher in the trabecular pattern of BCA, the morphologic pattern that can show overlap with cellular PA, increasing the value of this maker in this specific diagnostic challenge. In addition, this and other works have not demonstrated nuclear B-catenin expression in PA [14, 16, 17, 24].

Our work demonstrates the advantages of using an IHC panel in the differential diagnosis between cellular PA and BCA. According to our results, the IHC panel is more helpful when typical morphologic characteristics are absent. The use of three IHC markers that show opposite staining patterns in BCA and PA can be used to confirm the histological diagnosis. In our series, we found that up to 40% of morphologic diagnosis were erroneously made. Our work supports the idea that concise IHC panels can aid in the diagnosis of specific morphologic challenges in salivary gland pathology. In addition, other confirmatory tests, like PLAG1 gene rearrangement which is usually found in PA could further refine the histologic diagnosis. We consider that correct tumor classification will improve diagnostic precision and therefore, the prognostic information provided to the patient and clinicians.

## Conclusion

Given their morphologic overlap, the differential diagnosis between PA and BCA can be challenging. According to our results, when characteristic morphologic features are absent, the diagnosis of PA or BCA cannot rely on morphology alone. A substantial rate of diagnostic mistakes could be avoided by applying a GFAP, DOG1 and B-Catenin based IHC panel.

**Table 1:** Summary of cases included in the study. Cases that were reclassified after immunohistochemistry evaluation are highlighted. IHC results are dichotomized as stated in the text. The IHC evaluation performed by the expert pathologist is shown. F: Female, M: Male. B-cat: Beta Catenin, NA: Not Available

Case	Age, gender	Location	Morphologic diagnosis	GFAP	B-Cat	DOG-1	Final diagnosis	Clinical outcome (Years, outcome)
1	49 F	Parotid	BCA	**-**	**+**	**+**	BCA	2, no relapse
2	78 F	Parotid	BCA	**+**	**-**	**-**	PA	NA
3	57 F	Parotid	BCA	**+**	**-**	**-**	PA	NA
4	67 F	Parotid	BCA	**-**	**+**	**+**	BCA	NA
5	51 F	Parotid	BCA	**-**	**+**	**+**	BCA	7, no relapse
6	71 M	Palate	PA	**+**	**-**	**-**	PA	5, no relapse
7	68 F	Parotid	PA	**+**	**-**	**-**	PA	NA
8	75 F	Parotid	BCA	**+**	**-**	**-**	PA	NA
9	34 F	Palate	BCA	**+**	**-**	**-**	PA	9, no relapse
10	47 F	Parotid	PA	**+**	**-**	**-**	PA	3, no relapse
11	54 M	Parotid	PA	**+**	**-**	**-**	PA	2, no relapse
12	64 F	Parotid	BCA	**+**	**-**	**-**	PA	7, no relapse
13	62 F	Parotid	PA	**-**	**+**	**+**	BCA	3, no relapse
14	57 M	Parotid	PA	**+**	**-**	**-**	PA	3, no relapse
15	67 M	Parotid	BCA	**-**	**+**	**+**	BCA	NA
16	58 F	Palate	PA	**-**	**-**	**-**	PA	2, no relapse
17	51 M	Parotid	PA	**+**	**-**	**-**	PA	1, no relapse
18	55 M	Parotid	PA	**-**	**+**	**+**	BCA	1 no relapse

## Data Availability

Not applicable.

## Code Availability (software application or custom code)

Code used to run statistical analyses is available upon request to the corresponding author.
